# Engineering Electron Transfer Pathway of Cytochrome P450s

**DOI:** 10.3390/molecules29112480

**Published:** 2024-05-24

**Authors:** Jingting He, Xin Liu, Chun Li

**Affiliations:** 1 Key Laboratory for Green Processing of Chemical Engineering of Xinjiang Bingtuan, School of Chemistry and Chemical Engineering, Shihezi 832003, China; hejingting0829@163.com; 2 Department of Chemical Engineering, Tsinghua University, Beijing 100084, China; 3 Key Lab for Industrial Biocatalysis, Ministry of Education, Tsinghua University, Beijing 100084, China; 4 Center for Synthetic and Systems Biology, Tsinghua University, Beijing 100084, China

**Keywords:** cytochrome P450s, electron transfer, redox-partners, NAD(P)H regeneration, protein engineering, electrochemically or photochemically driven

## Abstract

Cytochrome P450s (P450s), a superfamily of heme-containing enzymes, existed in animals, plants, and microorganisms. P450s can catalyze various regional and stereoselective oxidation reactions, which are widely used in natural product biosynthesis, drug metabolism, and biotechnology. In a typical catalytic cycle, P450s use redox proteins or domains to mediate electron transfer from NAD(P)H to heme iron. Therefore, the main factors determining the catalytic efficiency of P450s include not only the P450s themselves but also their redox-partners and electron transfer pathways. In this review, the electron transfer pathway engineering strategies of the P450s catalytic system are reviewed from four aspects: cofactor regeneration, selection of redox-partners, P450s and redox-partner engineering, and electrochemically or photochemically driven electron transfer.

## 1. Introduction

Cytochrome P450s (P450s) are a superfamily of heme-containing enzymes named for their absorption band at 450 nm in the form of a carbon monoxide-bound complex and widely distributed in kingdoms of life. As the most versatile biocatalyst, P450s can catalyze C-H hydroxylation, C=C double bond epoxidation, N-, and S-oxidation, O-, N- and S-dealkylation, aromatic coupling, C-C bond breaking, and many other common and uncommon reactions [1,2,3,4,5,6]. P450s have highly regio-selective and stereospecific oxidation activity against a variety of substrates and are widely involved in the metabolism of various exogenous substances such as environmental compounds, antibiotics, and drugs [7], as well as in the biosynthesis pathways of endogenous molecules such as sterols and fatty acids [8,9,10,11]. Therefore, P450s play an important role in drug development [12], natural product biosynthesis, and biotechnology [13,14,15,16].

The typical catalytic system of P450 is an electron transfer system composed of the P450 enzyme, substrate, electron shuttle carrier (cytochrome P450 redox-partners, CRPs), electron donor (NAD(P)H), and oxidizer (O_2_). However, the large-scale application of P450s is limited by the poor stability of enzymes and the requirement for redox-partners or expensive cofactors. P450s and their redox-partners need to be organized in the right stoichiometry and orientation to ensure efficient electron transfer and support the P450s catalytic process. With more and more research on the structure and catalytic system of the P450 enzyme, P450s have been heterologously expressed in animal, plant, and microbial host cells, such as in the baculovirus-insect cell expression system [17], the tobacco osmotic transient expression system [18,19], and the prokaryotic/eukaryotic microbial expression system. Due to the unknown and complex nature, of the whole cell P450 catalytic system, many researchers have realized the catalysis of P450 by designing electron transfer pathways in vitro.

Both in vivo and in vitro catalytic systems have been limited by several technical bottlenecks, including low heterologous expression enzyme activity, the need for electron transfer partners, and the expensive cofactor NAD(P)H [20]. To solve these problems, strategies such as P450s mining and screening, host cell screening, redox-partner engineering, and NAD(P)H regeneration have been proposed. Herein, we reviewed the application of electron transfer engineering strategies in recent years from four aspects: cofactor regeneration, selection of redox-partners, P450 and CRP engineering, and electrochemically/photochemically driven electron transfer engineering.

## 2. Cytochrome P450s

The highly diverse P450s superfamily (with an amino acid sequence identity as low as 16% [21]) maintains a highly conserved 3D structural fold in which cysteine is fully conserved as the fifth (axial) ligand of heme iron and a catalytic cycle that is used together for O_2_ isomerization cleavage and the formation of highly active substances [22,23]. In most instances, the catalytic cycle of the P450s is based on the cleavage of dioxygen, resulting in the hydroxylation of the substrate and the formation of water [24]. Electrons are transferred from NAD(P)H to iron atoms in the heme groups of P450s by redox-partners [5].

### 2.1. Classification of P450s Systems

**Figure 1:**
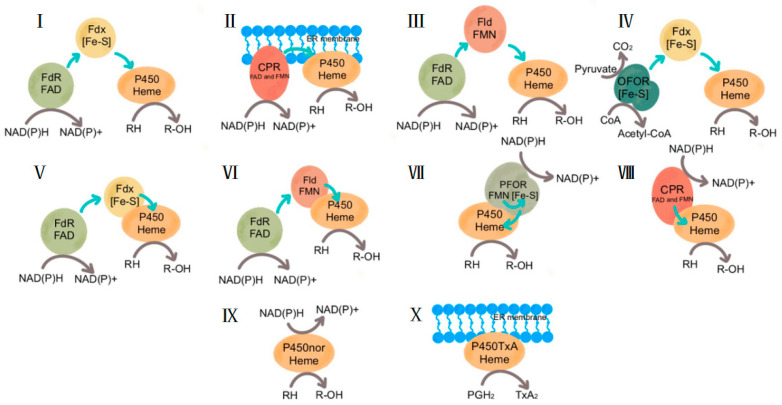
Schematic organization of different P450s systems. Abbreviations: FdR, ferredoxin reductase; FAD, flavin adenine dinucleotide; Fdx, ferredoxin; CPR, cytochrome P450 reductase; ER, endoplasmic reticulum; Fld, flavodoxin; FMN, flavin mononucleotide; OFOR, 2-oxo-acid-ferredoxin oxidoreductase; PFOR, phthalate family oxygenase reductase.

The P450s can be divided into ten categories [25,26], as shown in Figure 1, according to the redox-partner protein required by P450s in the catalytic reaction process. Classes I commonly found in bacteria and mitochondria consist of a P450 enzyme, a ferredoxin reductase redox-partner (FdR; cofactor flavin adenine dinucleotide (FAD)), and a small iron-sulfur protein-containing ferredoxin (Fdx). Classes II, present in eukaryotes, consist of a P450 enzyme and a membrane-bound cytochrome P450 reductase (CPR) as redox-partner, containing both FAD and flavin mononucleotide (FMN). Classes III: found in bacteria, the Fdx is replaced by a flavodoxin (Fld) containing FMN. Classes IV were first discovered in *Sulfolobus solfataricus*, where the FdR is replaced by 2-oxo-acid-ferredoxin oxidoreductase (OFOR). Classes V, first discovered in *Methylococcus capsulatus*, are fused with their redox-partners, Fdx. Classes VI, first discovered in *Rhodococcus rhodochrous*, are fused with their redox-partners, Fld. Class VII, first discovered in *Rhodococcus*, consists of an N-terminal P450 heme domain and a C-terminal reductase domain, consisting of an FMN-binding domain, an NADH-binding domain, and a [2Fe-2S] ferredoxin domain. Class VIII enzymes, first discovered in *Bacillus megaterium,* are self-sufficient enzymes, that contain binding sites for heme fused with CRP through a linker. Class IX is the only water-soluble eukaryotic P450 enzyme and can directly use NAD(P)H as an electron donor. Class X is independent of oxygen, NAD(P)H, and CRP however, uses acyl hydroperoxides as both substrate and oxygen donor.

### 2.2. P450 Catalytic Mechanism

In order to achieve the reductive activation of the inert O_2_ to the substrate mono-oxygenation, the vast majority of P450s interact with CRPs to obtain a reduction equivalent from NAD(P)H. In most instances, the NAD(P)H→CRP(s)→P450→Substrate electron transfer (ET) system is formed [27].

**Figure 2:**
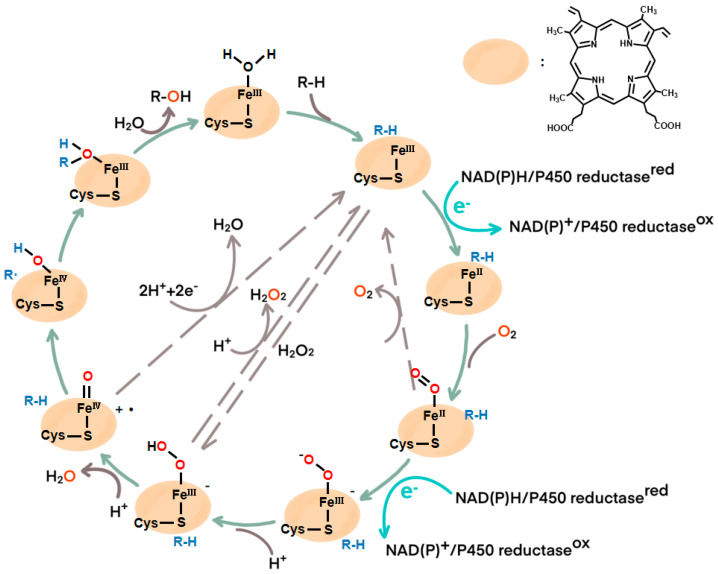
Schematic diagram of the P450 catalytic mechanism.

First, the substrate enters the reaction pocket to replace the bound water molecule at the active site, Fe^III^-OH_2_, interacting with Fe^III^ and binding the C-H bond to form the high-spin Fe^III^-RH. The CRP then transfers one electron from NAD(P)H to Fe^III^-RH, forming Fe^II^-RH. After that, it combines with molecular oxygen to form Fe^II^-O_2_, receives a second electron transferred from CRP, and produces a Fe^III^-OOH complex by protonation of protons obtained from the solution. The O-O bond of Fe^III^-OOH is then broken by a second proton from the solvent, releasing an H_2_O molecule to form the high-priced Fe^Ⅳ^=O, a cation that uses a radical mechanism to insert oxygen atoms into the substrate R-H bond to form Fe^Ⅳ^-OH. The substrate free radical dissociates from iron to produce the product, and a molecule of water returns to the Fe^III^ coordination, as shown in Figure 2.

Most P450s share a common monooxygenation mechanism, as mentioned above, which depends on NAD(P)H and CRPs. However, a few P450s (such as CYP152 peroxygenases) have evolved to directly utilize H_2_O_2_ as the sole oxygen and electron donor, forming Fe^III^-OOH complexes that catalyze fatty acids to hydroxylation products via the peroxide shunt pathway (dashed arrow in Figure 2) [28,29]. However, most P450s have a low H_2_O_2_ tolerance, which greatly limits their H_2_O_2_ shunt pathway [30]. Therefore, many researchers have made an effort to modify P450 monooxygenases into their peroxizyme by directed evolution or site-directed mutagenesis to simplify the common electron transfer chain and expand P450’s practical application [31,32]. Fan et al. developed a dual-functional small molecule (DFSM) strategy in which one end of DFSM binds to the P450BM3 protein with the anchoring group and the other end binds to the imidazolyl group. DFSM promoted the O-O cleavage of the adduct Fe^III^-OOH, which is beneficial to the formation of Fe^Ⅳ^=O of the active species, thus converting P450BM3 monooxygenase into peroxygenase [33]. Podgorski et al. modified selected residues within the I-helix of CYP199A4 to more closely resemble those of a natural peroxygenase and obtained a better mutant that functioned better at lower H_2_O_2_ concentrations and kept regio- and stereoselective hydroxylation ability [34].

## 3. Electron Transfer Pathway Engineering

**Figure 3:**
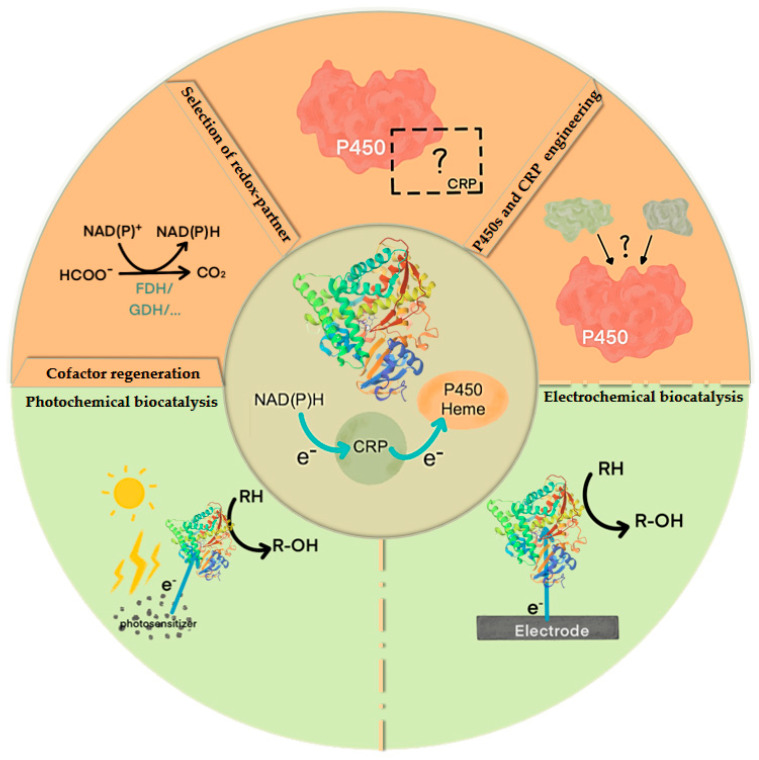
Strategies for engineering the electron transfer pathway of cytochrome P450s.

In general, a functional P450 system requires a redox-partner to transfer electrons from NAD(P)H to heme [35]. Therefore, in order to improve the catalytic efficiency of P450s, optimizing or reshaping the electron transfer pathway is an important approach, including the supply of electrons, the selection of CRPs, and enzyme engineering (Figure 3).

### 3.1. Cofactor Regeneration

In natural biocatalytic processes, cytochrome P450 enzymes catalyze a variety of oxidative transformations using the pyridine nucleotide NADH or NADPH as a cofactor [36]. However, due to the relatively low intracellular NAD(P)H concentration and the relatively expensive exogenous addition, the catalytic rate and application of P450s are greatly limited. Regeneration of NAD(P)H requires the transfer of one proton and two electrons (from the sacrificed hydride donor, such as glucose, formate, phosphite, or triethanolamine (TEOA)) to NAD(P)^+^. In general, the regeneration of cofactor NAD(P)H can be carried out by enzymatic, chemical, photocatalytic, and electrochemical methods [37,38]. The method of using enzymes to regenerate NAD(P)H is considered a favorable system due to its compatible reaction conditions, high selectivity, and high efficiency, and is widely used in practical industrial production [39].

The most widely used enzymes for cofactor regeneration in commercial processes are formate dehydrogenase (FDH) and glucose dehydrogenase (GDH), whereas phosphite (PDH), alcohol (ADH), and glucose 6-phosphate dehydrogenases (G6PDH) have been tested at laboratory scale [40,41]. Ma et al. [42] mutated formate dehydrogenase from *Candida dubliniensis*, and the catalytic efficiency of the mutant *Cd*FDH-M4 was improved 75-fold, with total cofactor turnover numbers (TTN) ranging from 135 to 986. Xu et al. [43] mutated the alcohol dehydrogenase from *Clostridium beijerinckii* to promote the NADPH coenzyme cycle, so that the conversion rate of the *Lk*ADH and *Cb*ADH reactions reached 100% within 6 h. Xiong et al. [44] obtained a self-contained NADPH cofactor regeneration system when ethanol dehydrogenase (ADH) and cyclohexanone mono-oxygenase (CHMO) were co-expressed in *E. coli*, and the maximum yield of ε-caprolactone was 0.80 mol/mol when the ADH/CHMO ratio was 0.34. Zhang et al. [45] conjured thioredoxin1 (Trx1) and thioredoxin reductase (TR) from *Thermus thermophilus* to NADPH-dependent oxidoreductases (alcohol dehydrogenase and cyclohexanone monooxygenase) to achieve NADPH regeneration.

Because the natural cofactor NAD(P)H is widely involved in various metabolic reactions in the cell, a change in its concentration in the cell will cause disturbances to cell life activities [46]. Therefore, unnatural cofactors such as nicotinamide cytidine dinucleotides (NCD) [47] and nicotinamide mononucleotides (NMN) were designed to be coupled to oxidoreductases [48].

### 3.2. Selection of Redox-Partner

Due to the difference in structure and catalytic system of P450s from different sources, their heterologous expression effect in microbial cells is completely different. In most prokaryotic microorganisms, the P450s catalytic system mediates the conformational change of ferredoxin (Fdx) through electrostatic interactions between Fdx, P450s heme, and reductase domains, thereby transferring electrons to P450s [49,50]. In eukaryotes, p450 is usually localized to the membrane structure via a terminal transmembrane domain, and electrons are transferred to the P450 heme domain via CPR [26,51,52]. Many P450s recombinants have been used in *E. coli* to synthesize a variety of chemicals. At the same time, yeast cells are generally considered to be the preferred system for heterologous expression of membrane-binding proteins due to their endoplasmic reticulum. The redox-partner, an essential component of most P450s systems, transfers two electrons from NAD(P)H sequentially to the heme-iron reaction center, activating oxygen to insert the C-H bond, a rate-limiting step in the catalytic cycle. However, little information is available about the homologous redox-partner of P450s [53], which has greatly limited the functional exploration and practical application of P450s [54]. Therefore, exploring and trying different redox-partners to construct various hybrid P450 electron transfer systems is the main strategy to improve the catalytic efficiency of P450. Prokaryotic microbial systems do not encode CRPs, so exogenous introduction of CRP is required to re-establish the function of P450s. Giang et al. [55] reconstructed the CYP108N12 catalytic system using cymredosin, a [2Fe-2S] redox chaperone of P450, to increase electron transfer rate (from 13 ± 2 to 70 ± 1 μM NADH/min/μM CYP108N12) and NADH utilization efficiency (so-called coupling efficiency from 13% to 90%).

Although eukaryotic microorganisms have endogenous CRPs, exogenous CRPs are often introduced to maximize the catalytic effect of P450s. For example, pairing CFS with plant-derived ATR1 increased yeast plant-derived nicotine production by more than 50 times compared to yeast endogenous CRP [56]. Therefore, exogenous CRPs are often introduced to improve the catalytic efficiency of P450s. Liao et al. [57] found that heterogeneously expressed recombinant protein MaCPR2 from mulberry leaves in yeast can use NADPH as an electron donor to reduce cytochrome c and ferricyanide and catalyze the formation of chlorogenic acid. Wang et al. [58] showed that after the integration of *Glycyrrhiza uralensis* Cb5 in the *S. cerevisiae* strain, which integrated CYP88D6, CYP72A154, β-amyrin synthetase, and the NADPH-cytochrome P450 reductase *POR1* gene of *Arabidopsis thaliana*, the triterpenoid acid titer was increased by eight times. Pairing with different CRPs often also makes a substantial difference in P450s activity [56,59,60]. The Fe_2_S_2_ Fdxs have been found to be more likely to provide high electron transfer efficiency for bacterial Class I P450s [61]. Liu et al. [54] compared three commonly used alternative redox-partners, SelFdx1499/SelFdR0978, Adx/AdR, and Pdx/PdR. SelFdx1499/SelFdR0978 has a higher electron transfer efficiency when interacting with P450 monooxygenase PikC, and their catalytic efficiency for YC-17 was the highest, reaching 99.1%. Istiandari et al. [62] found that mutation of the CRP gene *LjCPR1* in *Lotus japonicus* had no effect on product yield, while deletion of the CRP gene *LjCPR2-1* significantly reduced soybean saponin precursor yield. When constructing the P450s catalytic system, it is necessary to consider not only their species in addition to, their expression quantity. Excess CRP activity is toxic to microbial cells because it causes the overproduction of reactive oxygen species. Some studies have shown that the optimal ratio of P450s to CRP is about 12:1 [63]. Therefore, adjusting the expression levels of P450s and CRP appears to be critical for achieving higher P450s catalytic efficiency [64,65,66].

Of course, in the microbial production process, in addition to the P450s catalytic system that requires heterologous expression of rAedox-partners, there are also some self-sufficient P450s (Class VIII in Figure 1) that do not require additional redox-partners [67], in which the P450 heme domain is covalently bound to the reduction domain [68]. Such as the CYP116B subfamily [69], P450 BM3 [70], and P450 AZC1 [71]. The P450-CRP fusion arrangement promotes rapid electron transfer from NADPH to P450 heme. Due to the proximity and specific localization of their domains and the need for no additional redox-partners, they have excellent electron transfer systems [53]. Cha et al. [72] explored the optimal synergistic ratio of (+)-nootkatone production by considering the oxidation of membrane-anchored cytochrome P450/P450 reductase system (HPO/AtCPR) by establishing high-throughput screening technology and metabolic engineering, and the adjustment of the ratio of HPO/AtCPR increased the yield of (+)-nootkatone by two times in *Saccharomyces cerevisiae*.

### 3.3. P450s and CRP Engineering

**Figure 4:**
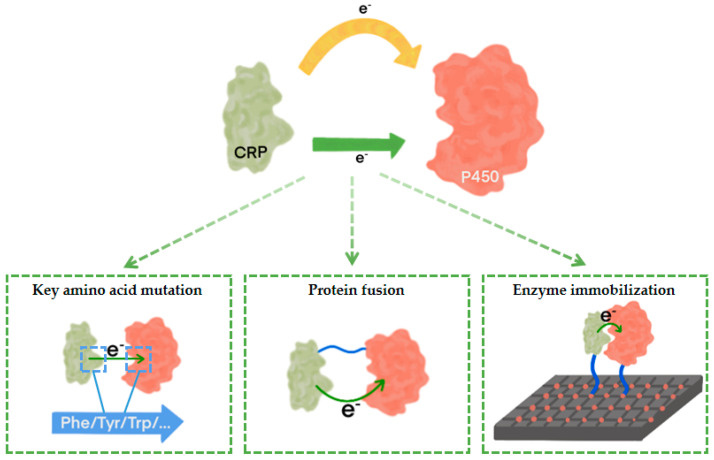
Schematic diagram of P450s and CRP engineering.

In the catalytic process of P450s, electrons from cofactors need to be transferred to the P450s heme domain through CRP. In order to improve the electron transfer rate of this step, the researchers engineered P450s and CRP from the protein structure (Figure 4).

#### 3.3.1. Key Amino Acid Mutation

P450s-mediated metabolism through proteins depends on the electron transfer chain—the interaction of a protein with its primary CRP. Traditionally, the P450s heme domain and CRP surface amino acid residues have been considered the main determinants of proper alignment in this interaction, in which hydrophobic interactions are also involved [73]. At present, many programs that can predict protein structure and calculate the protein-protein interaction have been designed for protein modification, such as AlphaFold2 (AF2), molecular dynamics (MD) simulation, QM/MM calculations, and so on. Cytochrome P450BM3 (CYP102A1) is a type VIII self-contained flavin hemoglobin with a heme and flavin domain in a polypeptide chain [74] that catalyzes subterminal oxidation of saturated and unsaturated fatty acids. Ivanov et al. [75] used AlphaFold2 and AlphaFold polymer (AFMultimer) programs to predict the three-dimensional structure of CYP102A1 (wild-type) protein monomer and homologous dimer and found that the homologous dimer F262 aromatic group is closer to heme. It increases the speed of electron transfer. Meng et al. [76] used molecular dynamics simulation and electron jump analysis of P450 BM3 mutants from *Bacillus megaterium* and showed that substituting the amino acid residues connecting FMN and heme iron with aromatic amino acids could increase the electron transfer rate and the catalytic effect by 13.9 times. Mutations of cytochrome P450 BM3, such as P450BM3 M7 mutants and P450BM3 M9 mutants, have improved electron transfer [77]. Velazquez et al. [78] expressed POR (OMIM:*124015, HNGC:9208) wild type and P228L variant in bacteria, and based on computer and in vitro studies, predicted that the change of proline to leucine might change the rigidity of protein, change the conformation of POR, and lead to a decrease in the rate of electron transfer to cytochrome c and thiazole blue tetrazole (MTT) significantly. Yu et al. [79] used a colorimetric high-throughput screening (HTS) system with DDA as the true substrate to conduct directed evolution and obtained a P450 mutant (R14R/D629G) with higher activity. Molecular docking analysis, kinetic parameter determination, and protein electrophoresis showed that the decrease in electron transfer distance between FMN and FAD caused by the D629G mutation is the main reason for the increased activity.

#### 3.3.2. Protein Fusion

CRPs, which are often fused to the C-terminal of P450s [80] to enhance their interactions and shorten the electron transfer chain, have been successfully applied in many model strains [81,82,83,84]. When constructing P450s-redox-partner fusion protein, it is important to consider the length of the linker, the amino acid content, and the position relative to P450s and CRP protein. Ligand length can affect the orientation, folding, correct conformation, and flexibility of enzymes [82]. Proline-rich rigid ligands [85,86] and glycine-rich flexible ligands [84,87] have been used more frequently to better control the spatial arrangement of enzymes and the structural isolation of fusion domains from surrounding amino acids and redox-partners, improving electron transfer [16,88]. Bakkes et al. [35] reported that the fusion efficiency of P450s and CRP with glycine-rich linkers was the highest (81.2%). Li et al. [89] used the GSTSSG linker to fuse CYP716A12 with ATR1 with N-terminal truncation, which increased the triterpene oleanolic acid (OA) yield to 129.9 mg/L.

#### 3.3.3. Enzyme Immobilization

Alternative techniques such as immobilization help to support the redox activity of P450 [81]. Park et al. [90] reported an electron channel strategy based on the application of CipB stent-protein, which allows efficient electron transfer between P450 and reductase by bringing these enzymes close together. The final lutein strain produced 218.0 mg/L of lutein in fed-batch fermentation. Wang et al. [91] constructed a protein scaffold multi-enzyme assembly to improve the coupling efficiency of the P450 system for efficient biosynthesis of daidzein from (2S)-naringenin.

### 3.4. Electro/Photo-Chemically Driven Electron Transfer Engineering

Despite the great potential of P450s, their dependence on NAD(P)H and redox-partners has limited their application in industry. In industrial processes, the production of NAD(P)H in cells is limited, exogenous addition is very expensive, and suitable redox-partners are difficult to find [92]. In order to overcome the dependence of cofactor NAD(P)H and redox-partner chaperones in the catalytic pathway of P450s, other approaches have been tried, such as electrochemical and photochemical methods on the P450s catalytic system [93,94]. In principle, the photochemical/electrochemical drive allows the conversion of luminous or electrical energy into chemical energy in the form of electron carriers, such as reduction cofactors, free radicals, or redox-partners, which are then used to drive the P450 catalytic reactions [95,96,97].

#### 3.4.1. Electrochemically Driven Electron Transfer Engineering

Since the catalytic cycle of P450s involves electron transfer processes [74,98], electrochemically driven P450s catalytic systems are considered to be a simple, clean, and pollution-free alternative to complex biological systems.

There are two main ways of electrochemically catalyzing P450s. One is to fix the enzyme on the surface of the electrode by surface modification. This way increases the electron coupling by increasing the contact area between the enzyme and the electrode surface, so that the electrons are transferred directly to the active center of the P450s [99,100,101,102,103,104], where redox-partners and the cofactor NAD(P)H are not necessary. Or transfer to the P450s active center via redox chaperones in fusion proteins. Castrignano et al. combined the heme domains of bacterial CYP3A4 and CYP116B5 with the reductase domain of BM3 to construct an efficient electron transfer chain [105]. Some studies have shown that the electrode surface characteristics, the porous structure of nanomaterials, and environmental conditions may affect the electron transfer efficiency [106,107,108]. These factors can control the orientation of P450s on the electrode, the electron loss of adverse reactions, and the amount of ROS, thus changing the efficiency of the electron coupling between heme and the electrode [109,110,111]. The most commonly used electrode surface modifications for the electrochemical catalysis of P450s include lipid film (didodecyldimethylammonium bromide (DDAB), polydienyldimethylammonium chloride, polydienyldimethylammonium bromide, polysodium p-styrene sulfonate), sol-gel methyltriethoxysilane of alkylmercaptan self-assembled monolayer (SAM), and composite materials based on various nanomaterials (gold nanoparticles, carbon nanomaterials), conductive polymers (polypyrrole, polythiophene, polyaniline, mercaptocarboxylic acid), etc. Electrochemical methods have high potential to investigate the different functions of P450s, including the study of the complex catalytic mechanisms of cytochrome P450s [103], as well as the design and development of new substrates, inhibitors, and modulators [112,113]. It has a promising application prospect in drug sensing devices, new drug search tools, and bioreactors [101]. Different P450s electrochemical catalytic systems will produce different catalytic effects, as shown in Table 1.

The other is to construct a P450 bioelectrocatalytic system (BES) [130], which can synergistically utilize electrons from electrodes and carbon source oxidation to achieve intracellular NAD(P)H regeneration and glucose oxidation through an electron shuttle carrier-mediated extracellular electron transfer (EET) pathway to promote P450 enzymatically catalyzed reactions. Common electron shuttles include riboflavin [131], methylviologen (MV) [132], and neutral red (NR) [130]. In BES with NR as electron shuttle carrier, the 7α-OH-DHEA yield catalyzed by CYP7B1 in *Saccharomyces cerevisiae* reached 288.6 ± 7.8 mg L^−1^, 2.4 times higher than that without EET (122.1 ± 3.7 mg L^−1^) [133].

#### 3.4.2. Photochemically Driven Electron Transfer Engineering

In the process of photochemically driven P450s catalysis, the photosensitizer and protein form a complex through covalent or non-covalent bonding using photogenerated electrons or electrons produced by the electron donor to drive the P450s-catalyzed reaction. Photosensitizers induce the direct or indirect activation of redox enzymes by photoinduced electron transfer, and light promotes electrons into higher energy states, while the holes vacated by excited electrons are filled at the expense of oxidation of the electron donor, such as water, triethanolamine (TEOA), or ethylenediaminetetraaceticacid (EDTA). Conduction band or triplet/singlet photoexcited electrons transfer to the redox centers of enzymes, such as P450 heme, flavin, and metal clusters. There are three main methods of light-driven electron transfer: (1) Active oxygen is generated in situ, and then P450 is catalyzed by the peroxide shingle pathway; (2) Redox-partners transfer electrons to the heme domain; and (3) Electrons are transferred directly to the heme domain [134]. Photosensitizers catalyze the redox conversion of the substrate by exchanging electrons at the active site of the enzyme [135,136]. The commonly used photosensitizers are protein photosensitizers (PS-I, PS-II, PSP), organic photosensitizers (porphyrin and Ru-pyridine) [137], and semiconductors (TiO_2_, carbon dot, CdS, g-C_3_N_4_, etc.) [138].

Girhard et al. [139] photoexcited flavin (riboflavin, flavin mononucleotide, or flavin adenine dinucleotide) with EDTA as an electron donor, and a photochemically driven CYP152A1 system to produce hydrogen peroxide in situ. Kim et al. [140] firstly used photosynthesis-derived electrons to immobilize P450-containing microsomes in spinach chloroplasts for light-driven bioreactors, and the chloroplasts of spinach were used for photosynthesis to produce NADPH for the oxidation of 7-ethyl coumarin (7-EC) to 7-hydroxycoumarin (7-HC) catalyzed by the CYP1A1-CRP fusion enzyme. Wang et al. [141] can effectively degrade amaranth by directly transferring photogenerated electrons generated by CdS to P450 through the electron transfer chain of *Shewanella* under the light-driven anaerobic condition. Lee et al. [95] used eosin Y as a photosensitive dye, TEOA as an electron donor, and [Cp*Rh (bpy) H_2_O] as an electron medium to drive NADPH regeneration and achieve O-dealkylation catalyzed by P450 BM3 variants (Y51F/F87A, BM3M2). Liu et al. [142] regenerated NADPH by combining engineered *Bacillus auriculare* with InP nanoparticles, which increased NADPH levels by 84 times.

Until Jensen recombinant PSI and purified CYP79A1 performed in vitro studies, the system relied on Fdx to transfer electrons from PSI to CYP79A1 without the involvement of NADPH and CRP, thus simplifying the previously proposed electron transfer pathway [96]. Natural flavin, as a photosensitizer, is often used to construct vision-driven whole cell platforms for P450s photocatalysis. The general applicability of light-driven flavin-mediated P450s photocatalysis systems was demonstrated in whole cells containing CYP1A1 [143], CYP1A2, CYP1B1, CYP2B4, and CYP3A4 [144,145,146]. In CdTe quantum dots (QDs) prepared by a covalent combination of CYP2D6 and QDs, electrons are transferred from the conduction band (CB) of CdTe quantum dots to CYP2D6 as a photocatalyst for drug metabolism [147]. When CYP119 is coupled with CeO_2_-3TiO_2_ semiconductor composite material, the coupling system shows higher photocatalytic reactivity than the uncoupled system under light [148]. In the hybrid P450 BM3 enzyme, the covalent linking of Ru (II)-diimide photosensitizer non-natural single cysteine residues enables rapid electron injection into the heme domain of several P450s for P450 oxidative functionalization on the trifluoromethylation substrate under visible light activation [149].

## 4. Conclusions and Future Perspectives

So far, many strategies have been developed to optimize the electron transfer process in P450s catalytic systems to improve the catalytic efficiency of P450s, and calculations such as molecular dynamics (MD) and quantum mechanics/molecular mechanics (QM/MM) are used to elucidate the electron transfer mechanism of P450s [150]. These include an adequate supply of electrons for the catalytic system, the selection and modification of P450s and CRP through rational/semi-rational design and directed evolution, the construction of photocatalysis and electrocatalysis systems, etc. Light as a free, green, and pollution-free energy, coupling P450s with the photosynthetic machinery of a plant, can use photogenerated electrons to drive the catalytic reaction [151], thus reducing the catalytic cost and improving the catalytic efficiency. With the pursuit of green, sustainable processes, the application of light or electricity to drive P450 catalysis may become mainstream. Combined with the advantages of biocatalysis and photoelectrocatalysis, the use of green energy for selective conversion has many advantages, including novel reactivity, high enantioselectivity, green synthesis, and high yield, which provide the basis for the application and development of P450s as bioreactors and biosensors [152]. Photo/electrically induced electron or energy transfer enables the synthesis method to complement the traditional dual electron transfer process for P450s or provide an orthogonal pathway for the development of new reactions. Enzymes can be regulated by directed evolution to exert control over intermediates, thereby inhibiting adverse reactions and providing high chemical and stereoselectivity. These strategies are combined to achieve green and efficient substance synthesis. In the future, the stability of P450s in vitro systems, the compatibility of CRP, electron transfer, and utilization efficiency will be the focus of P450s catalysis research.

## Figures and Tables

**Table 1:** Catalysis of P450s in different electrochemical systems.

Species	Electrodes	Km/μM	Substrate	Reference
CYP2C9	SPE ^1^/DDAB ^2^	45 ± 5	diclofenac	[114]
CYP2C9	CE ^3^/DDAB	3.03 ± 0.38	(S)-7-hydroxywarfarin	[115]
CYP2C9	GOLD/SAM	3	warfarin	[116]
CYP2C19	CE/DDAB	25.8 ± 2.0	4-hydroxyphenytoin	[117]
CYP2D6	CE/SPE	-	MDMA and α-PVP	[118]
CYP2D6	MWNT/[BMIM][PF6]-[TTF–TCNQ]/[BMIM][PF6]	5.52	Perphenazine	[119]
CYP2E1	SPE/DDAB	78 ± 9	chlorzoxazone	[120]
CYP3A4	SPE/DDAB/SLO ^4^	207 ± 2.5	erythromycin	[121]
CYP3A4	DDAB	70	erythromycin	[101]
CYP3A4	DDAB	-	-	[122]
CYP3A4	GCE/GO/DDAB	29.6 ± 4.1	phorate	[123]
CYP3A4	SPE/(PB-b-PDMAEMA/MWCNTs)	48 ± 8	diclofenac	[112]
CYP3A4	SPE/DDAB	10 ± 2	6β-hydroxycortisol	[124]
CYP19A1	GE/DDAB	4.2 ± 1.5	estrone	[125]
CYP55B1	PGE/GA/BSA	11.64 × 10^−3^	Nitric oxide reductase	[126]
CYP101	GC/SWCNT	-	-	[127]
CYP109D1	SPE/DDAB	-	myristic acid	[128]
P450 BM3	GCE/NH2-DMSN	244.82	testosterone	[129]

^1^ screen-printed electrodes. ^2^ didodecyldimethylammonium bromide. ^3^ graphite electrodes. ^4^ streptolysin O.

## Data Availability

No new data were created or analyzed in this study. Data sharing is not applicable to this article.
